# Inflammatory Cells Accelerated Carotid Artery Calcification *via* MMP9: Evidences From Single-Cell Analysis

**DOI:** 10.3389/fcvm.2021.766613

**Published:** 2021-12-06

**Authors:** Xiaobing Liang, Wanbing He, Hua Zhang, Dongling Luo, Zhengzhipeng Zhang, Aiting Liu, Jinkai Wang, Hui Huang

**Affiliations:** ^1^Department of Cardiology, The Eighth Affiliated Hospital, Sun Yat-sen University, Guangdong, China; ^2^Department of Cardiology, Sun Yat-sen Memorial Hospital, Sun Yat-sen University, Guangdong, China; ^3^Department of Medical Informatics, ZhongShan School of Medicine, Sun Yat-sen University, Guangdong, China

**Keywords:** single cell sequencing, vascular calcification, vascular smooth muscle cells, phenotypic modulation, MMP9, atherosclerosis, inflammation

## Abstract

**Background:** Vascular calcification (VC) is an important predictor of prognosis in atherosclerosis, the phenotypic transformation of vascular smooth muscle cells (VSMCs) is thought to be a process of VC. However, the implications and potential mechanisms for VSMCs phenotypic transition remain unknown.

**Methods:** To study the transformation of vascular smooth muscle cells (VSMCs) in the calcification early period, we analyzed single-cell sequencing data from carotid artery calcified core and paracellular tissue, based on the results of enrichment analysis and protein-protein interaction analysis. Upstream transcription factors were tracked and finally the results were validated using the MESA database.

**Results:** We successfully identified a subpopulation of inflammatory macrophage-like VSMCs and determined that MMP9 is an important factor in the phenotypic transformation of VSMCs. We found that RELA regulates MMP9 expression and that knockdown of RELA attenuated MMP9 expression and reduced the expression of BMP2 and the macrophage marker LGALS3 in vascular smooth muscle in inflammatory states, while serum levels of MMP9 correlated significantly with the inflammatory response.

**Conclusion:** This study reveals that the phenotypic transformation of VSMCs can be regulated by modulating MMP9, providing a new idea for the early treatment of VC.

## Introduction

Vascular calcification (VC) is an independent predictor for the prognosis in atherosclerotic patients. Despite the current advances in various treatments for atherosclerosis, including drug therapy and surgical resection, VC is still associated with increased mortality in patients (1). Various pharmacological treatments have been administered for arterial VC, including calcium channel blockers, inhibitors of the renin-angiotensin-aldosterone system, statins, vitamin K and others (2), but the results are unsatisfactory. vascular smooth muscle cells (VSMCs) play an important role in the process of arterial VC (3). It has been previously shown that VSMCs phenotypic transdifferentiation from contractile to osteogenic is a prerequisite for VC (4, 5) in the atherosclerotic setting, VSMCs undergo phenotypic modulation can develop into inflammatory macrophage-like cells with upregulated expression of LGALS3, or become 'synthetic' VSMCs (6). However, the specific regulatory processes involved in the regulation of VSMCs phenotypes have not been clarified. Since VC is irreversible and when it occurs, the vascular condition will not fully recover (7), prediction and prevention of VC is essential. Recent advances in single-cell transcriptomic sequencing have made comprehensive analysis of transcriptome expression of individual cells possible, providing the opportunity to identify cells into different states of transition (8). The use of single-cell sequencing to study the phenotypic transition of VSMCs in the calcification process has not been reported. Seurat is an algorithm that can integrate multiple single cell sequencing datasets to enable the classification of different cells based on their characteristics (9). By using Seurat and enrichment analysis, we can sort out subclasses of cells in samples from different sampling sites and explore the role of this class of cells in arterial VC.

In this study, we used a single-cell transcriptome sequencing database of entire calcified atherosclerotic core (AC) plaques and patient-matched proximal adjacent (PA) portions of carotid artery tissue from patients undergoing carotid endarterectomy. Macrophage-like cell subpopulations were identified by the t-distributed stochastic neighbor embedding (tSNE) algorithm (10), which revealed that the proportion of macrophage-like cells was most variable in the comparison between AC and PA. We further explored the biological functions of macrophage-like cells using enrichment analysis and found that they were associated with neutrophil activation. Protein interaction analysis was used to obtain that MMP9 was central to the overall protein regulatory network. Then, based on the differentially expressed genes in this subpopulation, we traced the common upstream of these genes and found that the expression of most differential genes was regulated by NFκB1 and RELA. Finally, we selected a high-throughput sequencing database associated with VSMCs with knockout of RELA genes and calculated the expression of related genes based on the inflammatory state. Induction of the inflammatory state in VSMCs with TNFα resulted in a decrease in the rise of MMP9, BMP2 and the macrophage marker LGALS3 following knockout of RELA. We determined that knockout of RELA reduces the expression of MMP9, which can affect phenotypic transition and calcification progression in VSMCs. The Multi-Ethnic Study of Atherosclerosis (MESA) examines disease characteristics in patients with cardiovascular disease across multiple regions and uses these risk factors to predict the progression of cardiovascular disease. The baseline examination 1 collected demographics, laboratory data and coronary computed tomography scans. To further define our results, we analyzed the relationship between serum MMP9 levels and clinicopathophysiological characteristics by using the MESA database. Significant associations were found between MMP9 and BMI, Agatston calcium score, C-reactive protein, and TNF-R1. This study could provide new ideas for the prevention and early intervention of arterial VC.

## Materials and Methods

### Data Collection

ScRNA-seq data from human carotid endarterectomy tissues and multiple high-throughput sequencing data were used for analysis in this study. ScRNA-seq data for a total of 51,721 cells from three human atherosclerotic calcified core plaques and their collateral tissues were obtained from Gene Expression Omnibus (GEO, http://www.ncbi.nlm.nih.gov/geo), registration number GSE159677 database, which contains 39,244 cells from the calcified core and 12,477 cells from the paratissue, based on Illumina NextSeq 500 with a read depth of 10x genomics. A total of 8 data from vascular smooth muscle cell samples with registration numbers GSM3175352, GSM3175352, GSM3175354, GSM3175355, GSM3175360, GSM3175361, GSM3175362, GSM3175363, from human embryonic stem cells with CRISPR / Cas9-mediated Gene edited human embryonic stem cells (hESCs) that differentiate into VSMCs.

### Processing of scRNA-Seq Data

A total of 51,721 cells from the calcified core and its paracrine tissue were included in this analysis. The Seurat software package in R with version number 3.2.0 was used for quality control, statistical analysis and exploration of the scRNA-seq data.

Supplementary Figure 1 lists gene expression before exclusions.

We excluded 6,711 low quality cells based on quality control criteria.

1. Exclusion of genes detected in <3 cells;
2. Excluded cells with <200 total genes detected;
3. Excluded cells with more than 6,000 total genes detected;
4. Cells with ≥10% of mitochondria-expressed genes were excluded.

Gene expression of the remaining 45,010 cells was normalized by using a linear regression model. Integrating data points were found for 6 samples and integrated (11). PCA was performed on the integrated results to identify usable data with a *p* < 0.05. Twenty initial PCs were used and the t-distributed stochastic neighbor embedding (tSNE) algorithm was applied for dimensionality reduction. A final cluster classification analysis was performed on all cells to obtain 20 clusters. Differential expression analysis was performed between genes with LogFC ≥ 0.5 within cell clusters using the limma package in R with version number 3.44.3 to identify marker genes for each cluster, adjusted for a *P* < 0.05. Subsequently, the marker genes were manually validated and corrected according to their compositional patterns by using the CellMarker database and existing experimentally proven cellular marker genes.

The corresponding genes used to annotate cell surface markers for cell clusters are listed in Supplementary Table 1.

### Enrichment Analysis and Protein-Protein Interactions Analysis

The external-gene-name was converted to entrezgeneid by using the biomaRt package (12) in R, version 2.44.1. The clusterProfiler package (13), version 3.16.1 in R, which automates the process of biomaRt classification and gene cluster enrichment analysis, was used to perform KEGG differential analysis (14) and GO differential analysis (15) of differential genes in cluster 7. The differential genes screened from cluster 7 are fed into PPI (https://string-db.org/) for analysis, which is used to construct protein-protein interaction networks and to analyse the molecular mechanisms of disease from multiple perspectives such as physical interactions or functional correlations. The PPI-derived data were subjected to statistical analysis using RStudio.

### Tracing Upstream Transcription Factors

Using TRRUST version 2 (https://www.grnpedia.org/trrust/), which contains data on human and murine transcriptional regulatory networks, the differential genes screened by cluster 7 were imported for analysis to obtain the transcription factors regulating the differential genes. Transcription factors and differential genes were counted and linked using Cytoscape software.

### High-Throughput Sequencing Data Processing and Statistics

All subgroups were divided into mainly WT and KO types, control and TNFα addition groups. The number of read counts of sequencing data was normalized using the DESeq2 package (16) with version number 1.28.1 in R. The gene expressions of RELA, MMP9, BMP2 and LGALS3 were extracted and analyzed using ANOVA as well as ordinary ANOVA test to assess whether the differences between gene expressions were statistically significant. And compare the mean on each column with the mean of every other column, with a confidence interval of 95%.

Meta data for 8 samples of VSMCs are presented in Supplementary Table 2.

### MESA Data Statistics and Analysis

MESA is a multi-ethnic observational cohort study that includes Caucasians, African, Americans, Chinese and Hispanics. A test from MESA in 2012 was used for this study, which included a test for serum MMP9 level. Institutional Review Board approval and informed consent of participants were obtained prior to the review (17). Blood pressure was classified according to “Hypertension by JNC VI (1997) criteria” and medical history. Body mass index (BMI) is calculated by dividing body weight (kg) by the square of height (m). The Agatston calcium score is used to determine the progression of coronary artery calcification (CAC) and is classified according to the presence or absence of CAC: no CAC (Agatston = 0) and CAC (Agatston > 0). C-reactive protein (CRP) rises in response to infection or tissue damage and can be classified according to the amount of level: normal (CRP ≤ 10), abnormal (CRP >10). Ankle-brachial index (ABI) is used to reveal vasoconstrictor function and can be divided into two types: normal (ABI ≥ 0.97) and abnormal (ABI < 0.97). The level of TNF-R1 was divided into two groups based on the median 1,223.

Statistical analysis was performed using IBM SPSS Statistics 25, and whether serum MMP9 level were associated with the clinicopathological characteristics was calculated using the Mann Whitney test or the Unpaired *t*-test. Subgroup analysis of the association between MMP9 levels and CAC progression using binary logistic analysis. Graphing was performed using GraphPad Prism 9.

## Results

### Analysis of scRNA-Seq Data Reveals High Cell Heterogeneity in Carotid Artery Calcification Core Tissues

**Figure 1:**
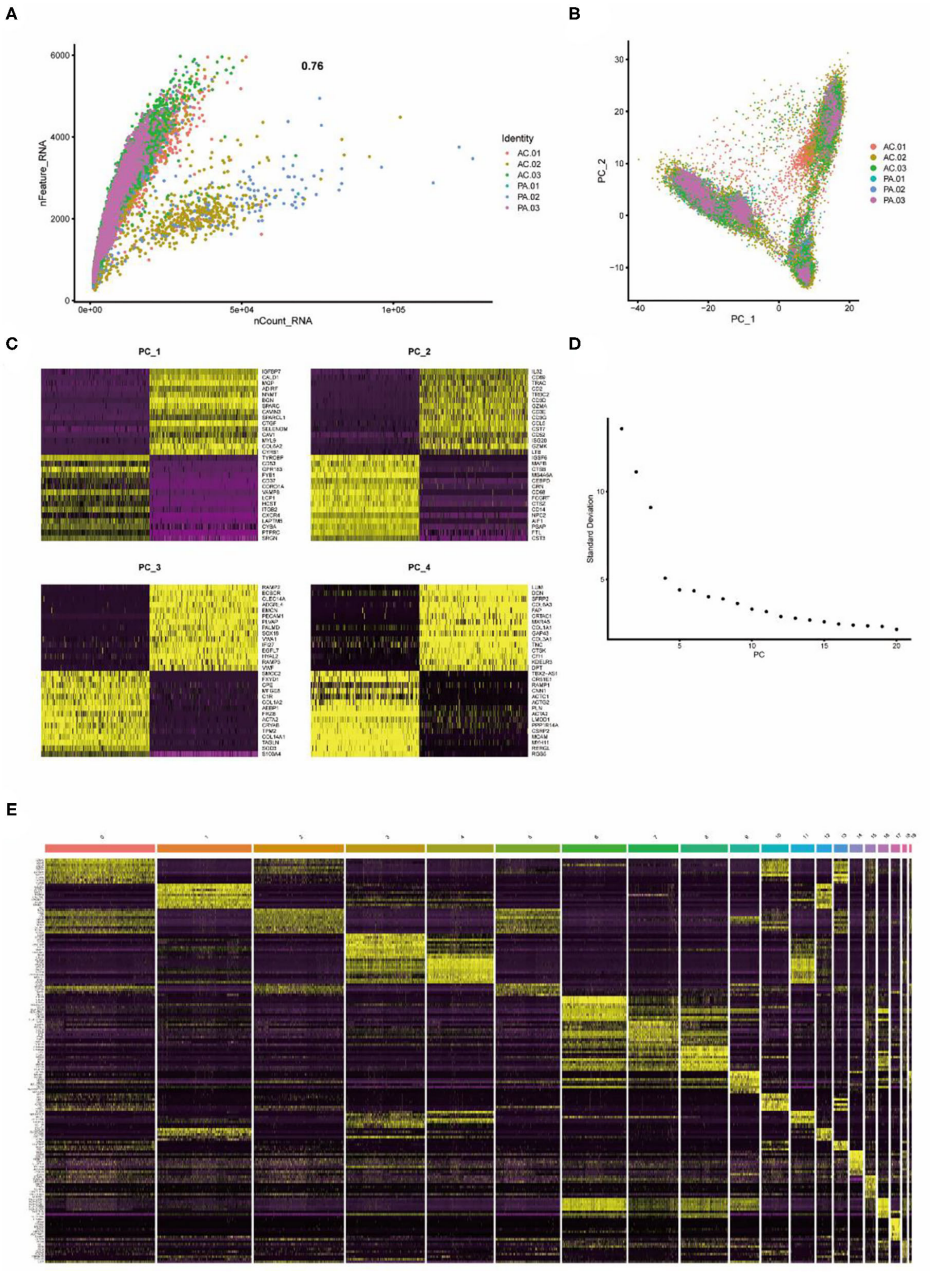
Twenty clusters identified based on single-cell RNA-Seq data reveal multiple cell subpopulations in calcified plaques. **(A)** The number of genes detected correlates significantly with the depth of sequencing. **(B)** PCA showing separation of cells in calcified plaques. **(C)** Differential genes in the first 4 PCs. **(D)** PCA identifies 20 PCs. **(E)** Differential analysis identifying the top 10 marker genes for each cell cluster is shown in the heat map. From yellow to purple indicates gene expression levels from high to low.

**Figure 2:**
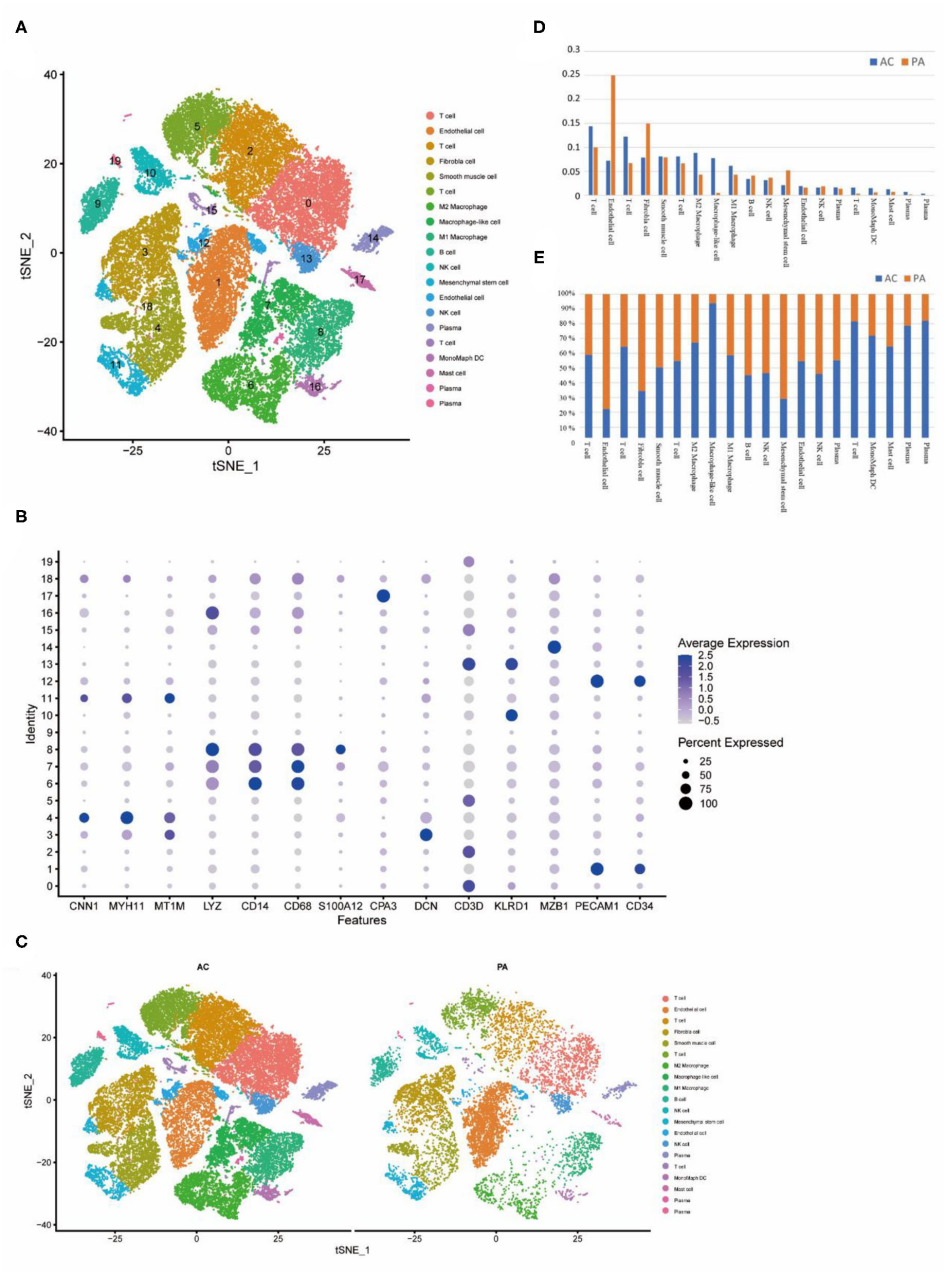
Cellular annotation of subpopulations of calcified core cells, grouped and shown in varying proportions. **(A)** The tSNE algorithm was applied to downscale the 20 PCs and divide the cells into 20 cell clusters. **(B)** Identification of markers for different cell clusters. **(C)** Different cell clusters have different proportions in AC and PA respectively. **(D)** Proportions of each of the 20 cell clusters in AC and PA respectively. **(E)** Variation in the proportion of the 20 cell clusters in AC and PA, with closer to 50% representing less variation.

Single-cell sequencing data from six carotid artery calcification core tissues were normalized according to quality control criteria, excluding cells with selection criteria, and the total number of cells in the six samples was reduced from 51,721 to 45,010, and the six samples were then combined. The number of genes detected correlated significantly with the depth of sequencing (Figure 1A). Principal component analysis (PCA) was performed on the tissue cells to identify available principal components (PCs) that were subsequently used to screen for relevant genes. There was some separation between tissue cells in the PCA results (Figure 1B). The differential genes in the heat map of the first four PCs were not identical (Figure 1C). Based on the results of the analysis, 20 PCs were selected for subsequent analysis (Figure 1D). Subsequently, using the t-distributed stochastic neighbor embedding (tSNE) algorithm, the carotid artery calcified core tissue was divided into 20 cell clusters (Figure 2A) and differential analysis was performed to obtain marker genes with logFC >0.5 in the 20 clusters (Figure 1E). These tissue cells could be divided into two groups, carotid artery calcified core (AC) and proximal adjacent to carotid artery (PA), based on different sampling locations.

### The Calcified Core and Paracellular Tissues Can be Classified into 20 Clusters

Based on the summary annotation of CellMarker and the currently known expression patterns of marker genes for each type of cell (Figure 2B), we have classified the 20 clusters as follows. Clusters 0, 2, 5 and 15 were labeled as T cells, contain a total of 14,981 cells; clusters 1, 12 were labeled as endothelial cells, containing a total of 5,964 cells; cluster 3 was labeled as fibroblasts, contained a total of 4,292 cells; cluster 4 was labeled as VSMCs, contained a total of 3,625 cells; clusters 6 were labeled as M2 Macrophage, contained a total of 3,499 cells; cluster 7 was labeled as macrophage-like VSMCs, contained a total of 2,722 cells; clusters 8 were labeled as M1 Macrophage, contained a total of 2,568 cells; cluster 9 was labeled as B-cells, contained a total of 1,602 cells; clusters 10 and 13 were labeled as NK cells, contained a total of 2,231 cells; cluster 11 was labeled as mesenchymal cells, contained a total of 1,287 cells; clusters 16 were labeled as M2 Monocyte, contained a total of 581 cells; cluster 17 was labeled as mast cells, contained a total of 509 cells.

In both groups, the proportion of different cell types varied (Figure 2C). In the AC group, the higher proportion were T cells, monocytes. In the PA group, the higher proportions were endothelial cells, fibroblasts (Figure 2D). In the comparison of the proportions of cells in the two groups, the largest percentage rise in the AC group was in cluster 7, which is macrophage-like smooth muscle cells, while inflammation-related cells such as T cells and monocytes all rose to varying degrees. In contrast, endothelial cells, fibroblasts and mesenchymal cells decreased substantially (Figure 2E).

### Enrichment Analysis Reveals That Cluster 7 is Highly Correlated With Inflammatory Activity

**Figure 3:**
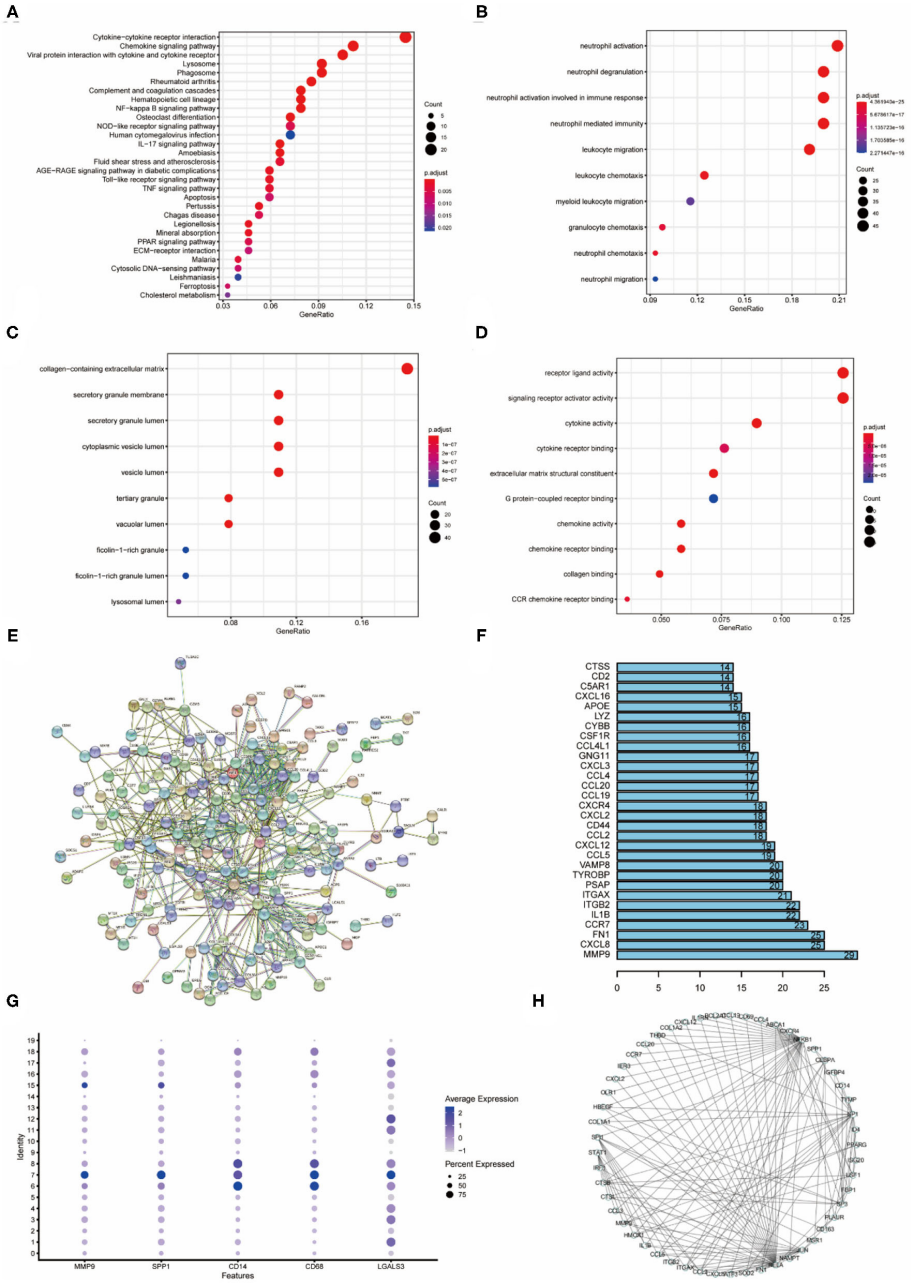
Enrichment analysis of cluster7, protein interactions, upstream transcription factors **(A)** KEGG enrichment analysis of cluster 7. **(B)** Biological process enrichment analysis of cluster 7. **(C)** Enrichment analysis of cellular component of cluster 7. **(D)** Enrichment analysis of molecular function of cluster 7. **(E)** Protein interaction network of cluster 7 differential genes. **(F)** Statistics of cluster 7 protein-interaction pairs. **(G)** Specific expression of cluster 7 genes. **(H)** Upstream transcription factor relationship map of cluster 7 differential genes.

Gene enrichment analysis (KEGG, BP, CC, MF) was performed on cluster 7 for the identification of relevant pathways, biological processes, cellular components, and molecular functions of macrophage-like VSMCs. The results showed that the cellular pathways of cluster 7 were mainly associated with cytokine receptor interactions and chemokine signaling (p < 0.001) (Figure 3A). The biological processes of cluster 7 were mainly associated with neutrophil activation, degranulation, migration, and immune response (*p* < 0.001) (Figure 3B). The main active cellular components of cluster 7 are the extracellular matrix and the granule membrane associated with secretion, and the granule lumen (*p* < 0.001) (Figure 3C). Cluster 7 active cellular functions are mainly for receptor ligands and signaling receptor activators (*p* < 0.001) (Figure 3D).

Protein interaction analysis of the differential genes of cluster 7 contained physical interactions and functional correlations between proteins (Figure 3E). In the protein-protein interaction network, there are 629 relational pairs, of which MMP9 is involved in 29 pairs, and other proteins that are more involved are CXCL8, FN1 and CCR7 (Figure 3F). MMP9, SPP1 (OPN), and LGALS3 were specifically expressed in cluster 7 (Figure 3G). The upstream transcription factors regulating differentially expressed genes in cluster 7 were identified based on the regulatory information of the genes, and the results showed that NFκB1 and RELA regulated the highest number of downstream (Figure 3H). Other transcription factors that regulated many downstream were SP1, JUN and SPI.

### Knockout of RELA Affects the Cellular Phenotypic Transition of VSMCs

**Figure 4:**
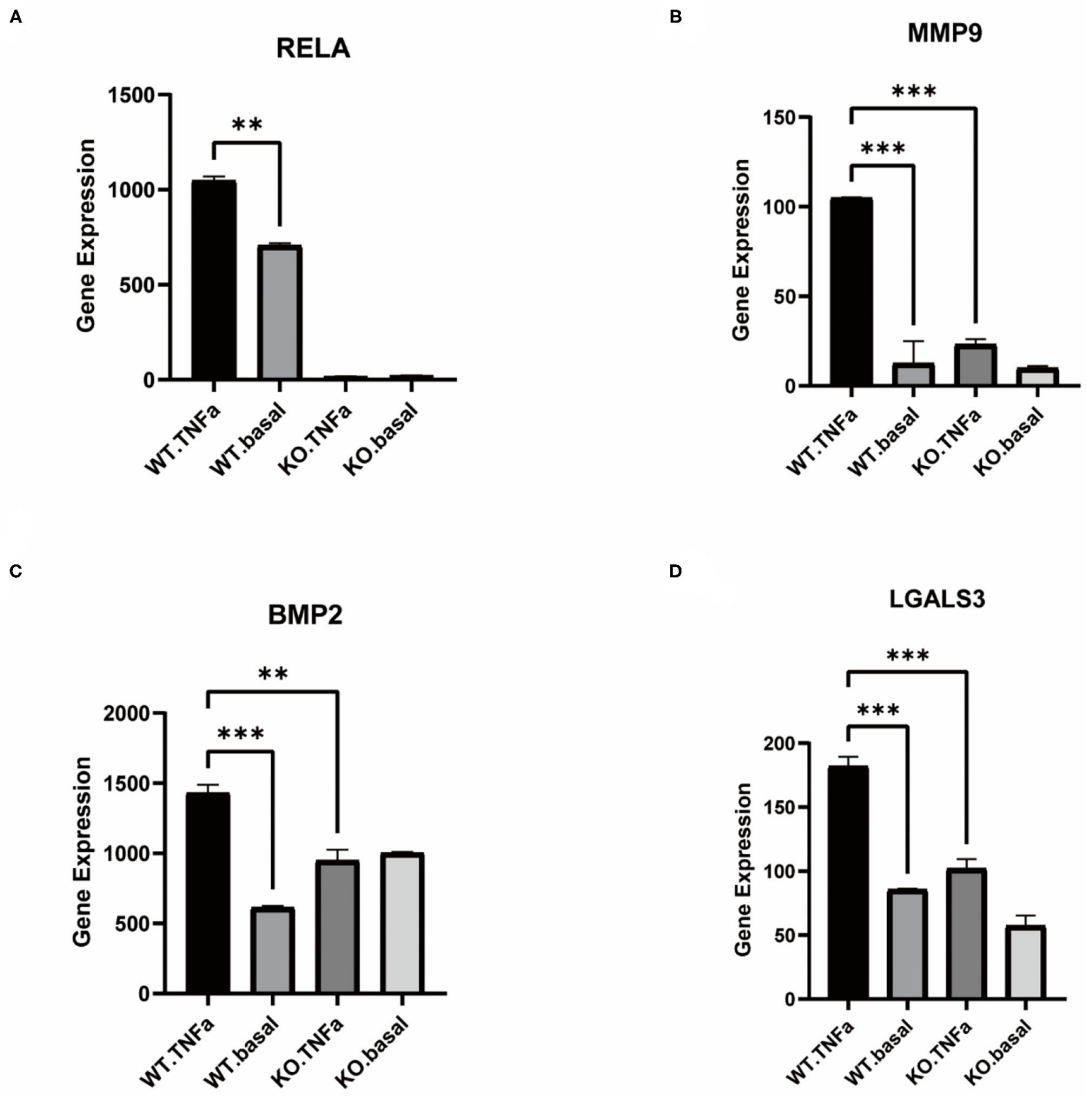
Comparison of gene expression in VSMCs before knockdown of RELA and after knockdown. **(A–D)** Expression of RELA, MMP9, BMP2 and LGALS3 in control and TNFα-added groups after knockdown vs. before knockdown, respectively. ^**^P < 0.01, ^***^P < 0.001.

TNFα induced an inflammatory response in the cells, and the addition of TNFα to WT-type VSMCs resulted in a rise in RELA expression, whereas it was not expressed in the KO-type VSMCs with RELA knocked out (Figure 4A). MMP9 expression was significantly higher in the inflammatory response, whereas knockdown of RELA resulted in a significantly lower rise in MMP9 than the WT-type (Figure 4B). Similarly, BMP2 expression rose in the inflammatory response but did not change significantly in the KO-type (Figure 4C). In contrast, the macrophage phenotype marker gene LGALS3, which was significantly elevated in the WT type stimulated with TNFα, had a reduced rise after knockdown of RELA compared to the WT-type (Figure 4D).

### Association Between Baseline Characteristics of MESA Participants and MMP9

A total of 999 participants were tested for serum MMP9 levels and 57.0% of the population were female with a mean age of all was 59.35 years at baseline (Table 1). Of the study population, 408 (40.8%) participants had hypertension and 716 (71.6%) had a BMI at overweight levels. Of these, 554 (55.4%) had no CAC (Agatston=0),445 (44.5%) had at least moderate CAC. In contrast, 927 (92.7%) had an ABI at or above 0.97 and only 0.72% had <0.97. In the serum level test, 97 (0.97%) people had CRP >10mg/L and 50% had TNF-R1 levels over 1223pg/mL. There was no significant difference in the effect of age, hypertension and ABI on MMP9 levels.

**Table 1:** Association between serum MMP9 level and the clinicopathological characteristics.

	**N**	**Mean ± SD**	***P*-value**
**Age (years)**			
≤60	579	242.98 ± 5.65	0.54
>60	420	248.58 ±7.38	
**Gender**			
Female	570	255.07 ± 6.32	0.035
Male	429	232.40 ± 6.25	
**Hypertension**			
No	591	240.87 ± 5.65	0.238
Yes	408	251.80 ± 7.40	
**BMI (kg/m** ^2^ **)**			
<25	282	232.29 ± 8.20	0.024
≥25	716	250.02 ± 5.37	
**Agatston calcium score**			
0	554	236.46 ± 5.59	0.031
>0	445	256.37 ± 7.33	
**C-reactive protein (mg/L)**			
≤10	901	236.68 ± 4.48	<0.0001
>10	97	322.40 ±18.60	
**Ankle-brachial index**			
<0.97	72	280.47 ± 20.57	0.086
≥0.97	927	242.6 ± 4.58	
**TNF-R1 (pg/mL)**			
<1,223	499	214.31 ± 4.99	<0.0001
≥1,223	500	276.3 ± 7.25	

**Figure 5:**
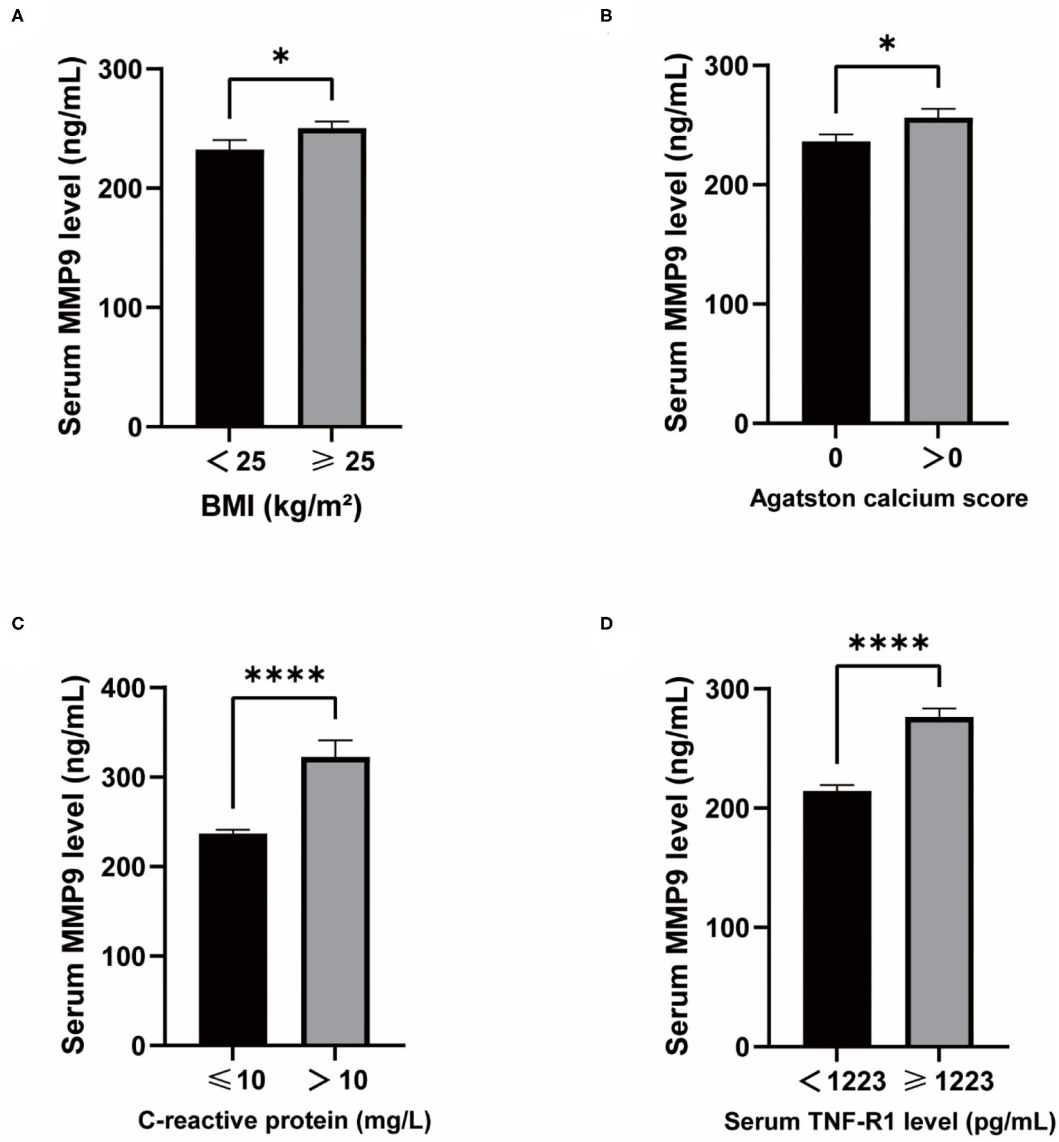
Differences in serum MMP9 levels and clinical physiopathological characteristics. **(A)** MMP9 levels in BMI ≥25 vs. BMI <25. **(B)** MMP9 levels in Agatston calcium score = 0 vs. Agatston calcium score > 0. **(C)** MMP9 levels in CRP ≤ 10 vs. CRP >10. **(D)** MMP9 levels in TNF-R1 <1,223 vs. TNF-R1 ≥1,223. ^*^P < 0.05, ^****^P < 0.0001.

MMP9 levels were higher in overweight than normal individuals (Figure 5A) and higher in those with CAC than in those without CAC (Figure 5B). CRP associated with infection and injury in the organism had a significant effect on serum MMP9 levels (Figure 5C). TNF-R1 plays a role in cellular inflammation and there was a significant difference in serum MMP9 levels between the two groups divided by a median of 1,223 pg/mL, with higher serum MMP9 levels in those with high TNF-R1 levels (Figure 5D).

To explore the specific populations at increased risk of CAC progression due to altered MMP9 levels, we conducted subgroup analyses based on traditional risk factors (Table 2). We found that the positive association between MMP9 levels and CAC progression was significant in females (OR 1.002; 95%CI 1.001–1.003; *P* < 0.0001).

**Table 2:** Subgroup analysis of the association between MMP9 level and CAC progression.

**Subgroups**	**OR**	**95%CI**	***P-*value**
**Age, years(%)**			
≤ 64 (69.6)	1.002	1.000–1.003	0.008
>64 (30.4)	1.001	1.000–1.003	0.02
**Sex(%)**			
Female (57.1)	1.002	1.001–1.003	<0.0001
Male (42.9)	1	0.999–1.002	0.569
**BMI, kg/m**^2^ **(%)**			
≤25 (28.2)	1.001	1.000–1.003	0.031
>25 (71.8)	1.001	1.000–1.002	0.043
**Hypertension (%)**			
No (59.2)	1.001	1.000–1.003	0.042
Yes (40.8)	1.001	1.000–1.002	0.063

## Discussion

Our study shows that macrophage-like cells transformed from VSMCs are involved in VC progression and that these cells are mainly associated with inflammatory responses. MMP9 plays an important role in the phenotypic transition of VSMCs and is regulated by RELA.

The phenotypic changes in VSMCs during the calcification process were often investigated using various chemicals or growth factors for experiments. A number of *in vitro* studies have found increased or decreased expression of smooth muscle cell markers, including upregulation of the macrophage marker LGALS3 (6), and the resulting differentiation of VSMCs into multiple subpopulations. In an *in vivo* study, Wirka et al. (18) proposed that VSMCs regulated by phenotypic alterations would change from contractile VSMCs to fibroblast-like cells along a continuous trajectory.

In this study, we found that macrophage-like cells were highly variable in calcified core plaques and were associated with inflammatory responses. These findings were based on a single cell transcriptome sequencing database and we validated the results using data from VSMCs with knockout RELA. As there are multiple cells in the calcified core plaques, we classified all cells into 20 cell clusters. In addition to the macrophage-like cells studied in this experiment, there were T cells, endothelial cells, fibroblasts, VSMCs, monocytes, B cells, NK cells, mesenchymal cells, and mast cells, all of which are consistent with the composition of the vasculature. In addition to the upregulation of LGALS3 expression in macrophage-like cells, the macrophage markers CD14 and CD68 were also upregulated, as were bone bridging protein (OPN) and bone morphogenetic protein 2 (BMP2), which are associated with calcification.

The results of the enrichment analysis showed that macrophage-like cells are involved in neutrophil activation and migration mainly through cytokine receptor interactions. To further explore the biological processes of macrophage-like cells, we analyzed the protein-protein interaction network and found that MMP9 was associated with the action of numerous proteins. The matrix metalloproteinase (MMP) family is involved in the breakdown of the extracellular matrix in numerous physiopathological processes and plays an important role in leukocyte migration. NFκB and RELA are transcriptional regulators that modulate the intracellular expression of MMP9 (19), and their inappropriate activation can lead to various inflammatory conditions. Previous experiments have demonstrated that TNFα action activates NFκB to promote the osteogenic transformation of VSMCs (20), however the exact process is not yet known.

By analyzing high-throughput sequencing data, we found that the expression of MMP9 in VSMCs could be reduced by knocking out RELA. In contrast, VSMCs induced by the addition of TNFα could transdifferentiate into macrophage-like cells, and the macrophage marker LGALS3 decreased dramatically after knockout of RELA. BMP2 induces cartilage and bone formation and plays an important role in the calcification process of VSMCs, and BMP2 expression rises in the inflammatory response and promotes calcification of VSMCs, while BMP2 expression after knockout of RELA did not change significantly.

This suggests that RELA may regulate the phenotypic transition and calcification process of VSMCs in the inflammatory response through MMP9. MMP9 increases the density of inflammatory infiltration, accelerates vascular injury and regulates the entry of monocytes and T cells into the vessel wall (21), and inhibition of MMP9 expression protects the vascular intima and reduces the inflammatory response.

In our derived results, inhibition of MMP9 inhibited the conversion of VSMCs into macrophage-like cells, delayed bone and cartilage differentiation and affected the formation of atherosclerotic calcified core plaques. The results of the MESA data analysis also demonstrate that MMP9 in plasma is associated with an inflammatory response, and that MMP9 levels are elevated to varying degrees in people with overweight and CAC.

A potential limitation of our study is that an animal model of arterial vascular calcification has not been used to further validate the specific effects of MMP9 on the transdifferentiation of VSMCs. Although an association between MMP9 and some of the clinical physiopathological features was clearly established, a causal relationship could not be demonstrated.

## Conclusions

Using scRNA-seq and high-throughput RNA-seq data, we found that inflammation can affect the transdifferentiation of VSMCs into macrophage-like cells, and that MMP9 is an important factor in this process. The role of MMP9 in promoting atherosclerotic plaque calcification was validated based on the MESA database. This study highlights the impact of MMP9 in early vascular calcification, as well as providing new ideas for improving prognosis in patients with atherosclerosis.

## Data Availability Statement

The datasets presented in this study can be found in online repositories. The names of the repository/repositories and accession number(s) can be found in the article/Supplementary Material.

## Author Contributions

XL is responsible for data collection and analysis, as well as article writing. HZ and DL are responsible for data collection. WH, ZZ, JW, and AL are responsible for the revision of the article. HH is financial support and revision of the article. All authors contributed to the article and approved the submitted version.

## Funding

This work was supported by National Natural Science Foundation of China (8201101103, 82073408, 81870506, 81670676, and 81422011), project of Traditional Chinese Medicine in Guangdong province (20201062), Basic Research Project of Shenzhen Science and Technology Innovation Committee (JCYJ20180306174648342 and JCYJ20190808102005602), Shenzhen Futian District Public Health Research Project (FTWS2019003), and Shenzhen Key Medical Discipline Construction Fund (SZXK002).

## Conflict of Interest

The authors declare that the research was conducted in the absence of any commercial or financial relationships that could be construed as a potential conflict of interest.

## Publisher's Note

All claims expressed in this article are solely those of the authors and do not necessarily represent those of their affiliated organizations, or those of the publisher, the editors and the reviewers. Any product that may be evaluated in this article, or claim that may be made by its manufacturer, is not guaranteed or endorsed by the publisher.
