# Supplementary material for: Inflammatory Cells Accelerated Carotid Artery Calcification via MMP9: Evidences From Single-Cell Analysis
Source: Front Cardiovasc Med. 2021 Dec 6;8:766613. doi: 10.3389/fcvm.2021.766613 (PMC8685327; doi:10.3389/fcvm.2021.766613)
Supplement: Supplementary file 1 [file Data_Sheet_1.DOCX]

Original data links

GSE159677:

<https://www.jianguoyun.com/p/DYvjcAQQ5_LmCRi2_osE> <https://www.ncbi.nlm.nih.gov/geo/query/acc.cgi?acc=GSE159677>

Codes used to analyze GSE159677: <https://www.jianguoyun.com/p/DRnoSvsQ5_LmCRiE_4sE>

GSE115311(Includes GSM3175352, GSM3175352, GSM3175354, GSM3175355, GSM3175360, GSM3175361, GSM3175362, GSM3175363):

<https://www.jianguoyun.com/p/DTqVECwQ5_LmCRiH_4sE>

<https://www.ncbi.nlm.nih.gov/geo/query/acc.cgi?acc=GSE115311>

Codes used to analyze GSE115311: <https://www.jianguoyun.com/p/DYEu01gQ5_LmCRiS_4sE>

MESA database: <https://www.jianguoyun.com/p/DUUSNkEQ5_LmCRiT_4sE>

MESA datadictionary: <https://www.jianguoyun.com/p/DV4wo3UQ5_LmCRiW_4sE>
