# Supplementary material for: Inflammatory Cells Accelerated Carotid Artery Calcification via MMP9: Evidences From Single-Cell Analysis
Source: Front Cardiovasc Med. 2021 Dec 6;8:766613. doi: 10.3389/fcvm.2021.766613 (PMC8685327; doi:10.3389/fcvm.2021.766613)
Supplement: Supplementary file 3 [file Data_Sheet_3.PDF]

Supplementary Material

SUPPLEMENTARY TABLES AND FIGURES

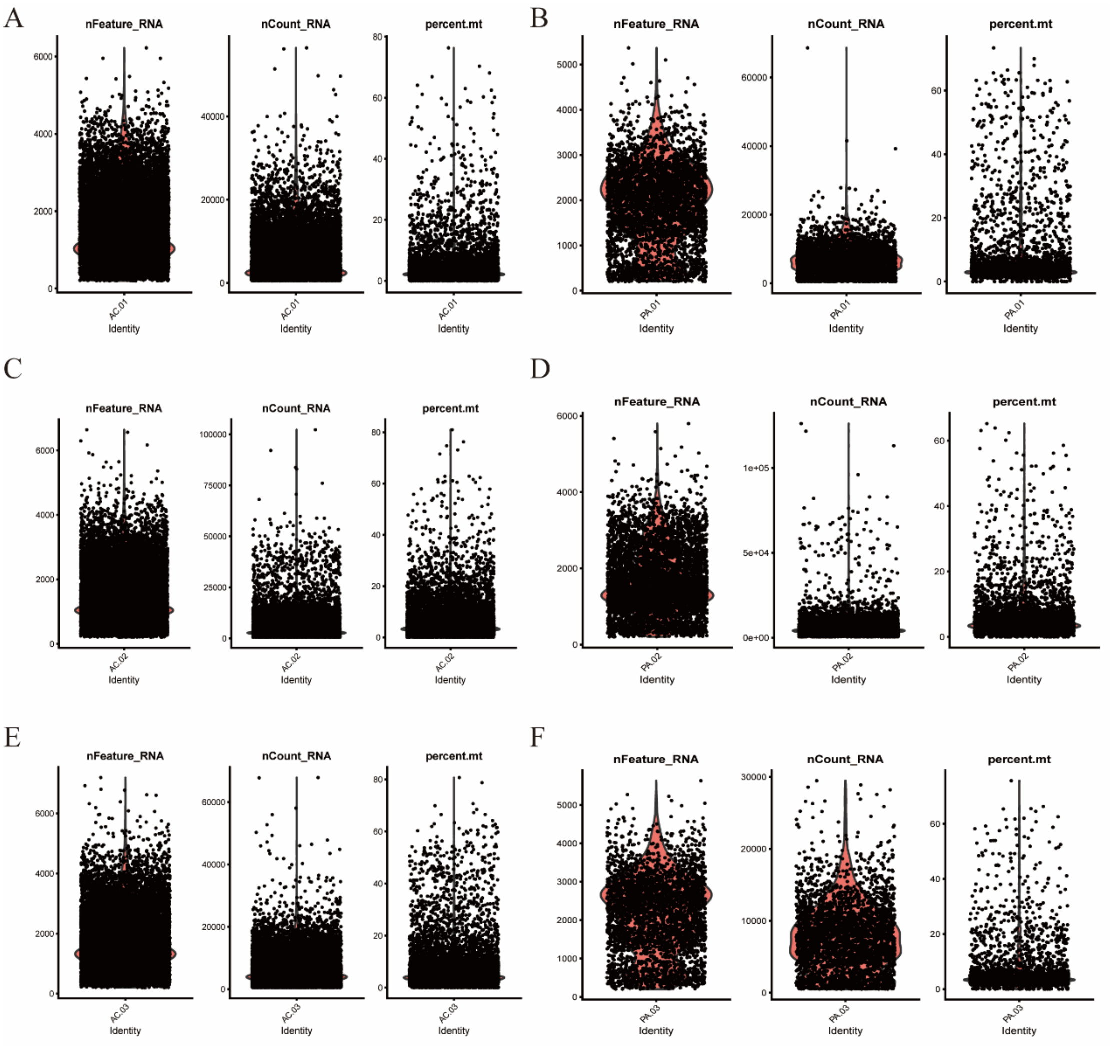

Figure S1. Number of RNA detections, RNA expression, and percentage of mitochondrial expression in 6 samples.

(A-B) Number of RNA detection, RNA expression, and percentage of mitochondrial expression in AC and PA tissues of patient 01.

(C-D) Number of RNA detections, RNA expression, and percentage of mitochondrial expression in AC and PA tissues of patient #02.

(E-F) Number of RNA detection, RNA expression and percentage of mitochondrial expression in AC and PA tissues of patient No. 03.

Table S1. Markers for identifying cluster cell type

cluster	cell type	marker
0	T cell	CD3D
1	Endothelial cell	CD34,PECAM1
2	T cell	CD3D
3	Fibrobla cell	DCN,MT1M
4	Smooth muscle cell	CNN1,MYH11,MT1M
5	T cell	CD3D
6	M2 Macrophage	CD14,CD68,LYZ,CD163
7	Macrophage-like cell	CD14,CD68,LYZ,LGALS3
8	M1 Macrophage	CD14,CD68,S100A12,LYZ,CXCL10
9	B cell	CD79A
10	NK cell	KLRD1
11	Mesenchymal stem cell	MT1M,CNN1,MYH11
12	Endothelial cell	CD34,PECAM1
13	NK cell	KLRD1,CD3D
14	Plasma	MZB1,CD79A
15	T cell	CD3D
16	MonoMaph DC	LYZ,CD14
17	Mast cell	CPA3
18	Plasma	CNN1,MYH11,MZB1,CD14
19	Plasma	CD3D,CD79A

Table S2. GEO database grouping

GEO	GeneType	treatment	CellType
GSM3175352	WT	TNFa	VSMC
GSM3175353	WT	TNFa	VSMC
GSM3175354	WT	basal	VSMC
GSM3175355	WT	basal	VSMC
GSM3175360	KO	TNFa	VSMC
GSM3175361	KO	TNFa	VSMC
GSM3175362	KO	basal	VSMC
GSM3175363	KO	basal	VSMC
